# MDMA-assisted therapy: challenges, clinical trials, and the future of MDMA in treating behavioral disorders

**DOI:** 10.1017/S1092852925000057

**Published:** 2025-01-30

**Authors:** Steve O’Brien, David Nutt

**Affiliations:** 1 Department of Brain Sciences, Imperial College London, London, UK; 2 Department of Brain Sciences,Faculty of Medicine, Imperial College London, London, UK

**Keywords:** Behavioral disorders, drug regulation, FDA, MDMA-assisted therapy, mental health, novel therapeutic innovations, PTSD, psychedelic research, psychotherapy

## Abstract

This chapter explores the complex and controversial path of MDMA-assisted therapy (MDMA-AT) for treating post-traumatic stress disorder (PTSD) and other behavioral disorders. It covers MDMA’s history from research to recreation to medicine, the pivotal trials, and the challenges faced by researchers. Despite recent setbacks for the clinical application of MDMA, the chapter argues that it holds potential for transforming psychiatry and discusses the uncertain future amidst ongoing debates over ethics, methodology, and political influence.

## Introduction to MDMA

Originally synthesized in 1912 by Merck chemist Anton Köllisch as an appetite suppressant,^1^ MDMA, or 3,4-methylenedioxymethamphetamine lay dormant for decades until its rediscovery by Alexander Shulgin in the 1970s.^2^ Following its initial patent submitted in the winter of 1912, MDMA remained unexplored in animal or human trials during its initial years at Merck. It wasn’t until decades later, in 1927 and 1952, that the company’s chemists conducted rudimentary pharmacological assessments, yet even then, there was no evidence of MDMA being tested on humans until 1960.^3^ While Köllisch met his untimely demise in the First World War, Shulgin’s famous approach to self-experimenting with psychoactive compounds unveiled MDMA’s remarkable ability to induce feelings of empathy, openness, and emotional closeness.^4^

In a poetic twist of fate, this compound, discovered in the shadow of a world soon to be torn apart by war, now emerges as a potential ally in the battle against the psychological scars left by trauma.

These effects sparked interest among psychotherapists for their potential to enhance communication and introspection. One of the first therapists to identify this potential was Shulgin’s wife, Ann, who used MDMA to assist in marriage counseling. Following several successful sessions, she described it as “penicillin for the soul”, before naming it “Empathy.” This label would subsequently be morphed into the name “Ecstasy”, as the compound moved from medicine to the club world of rave music and the sweaty euphoria invoked on dance floors across the world.

MDMA’s rise was catalyzed in the early 1980s when Michael Clegg, a former Catholic priest, introduced it to the Dallas club scene under the name “Therapy.” Its success there led Clegg to former marijuana distributor Bob McMillen, proposing to sell the drug on the West Coast. After McMillen sampled MDMA’s euphoric effects, he agreed—insisting on rebranding from the “corny” “Therapy” to “Ecstasy”, the name of the album he listened to during his first experience. Within 4 days, McMillen had sold 5000 units of rebranded Ecstasy.^5^ McMillen’s large-scale distribution marked MDMA’s transition from a therapeutic tool to a recreational drug, rapidly spreading through underground networks. Despite its 1985 criminalization, Ecstasy flourished in the rave/club scenes of the late 1980s and 1990s, becoming emblematic of the era’s dance culture.

The dawn of the 1980s cast a shadow over MDMA’s burgeoning acceptance, as the spectre of prohibition loomed large. Governments, gripped by fear and misunderstanding, cracked down on the booming rave culture, viewing MDMA as an agent of chaos rather than a potential ally in healing. It was a narrative all too familiar, echoing the vilification of LSD in the countercultural movements of the 1960s. Following obtaining an initial sample in Chicago in the 1970s, the Drug Enforcement Administration (DEA) spent 10 year examining the potential for MDMA abuse. On July 27, 1984, based on its chemical and pharmacological similarity to MDA (3,4-Methylenedioxyamphetamine—another of Shulgin’s empathogens, already banned), together with the propensity for self-administration, MDMA was placed into Schedule I in the United States.^6^ Other countries would soon follow suit.

The UK’s prohibition of MDMA followed a complex and arguably surprising path. In 1977, MDMA became prohibited through “proactive prohibition”—the first generic legislation of its kind—via a broad-ranging amendment to the Misuse of Drugs Act 1971.^7^ This type of legislative control would later be revived to address new psychoactive substances (NPS). Despite a lack of evidence regarding prevalence or associated problems, MDMA was one of several hundred chemicals banned in the 1977 amendment.^8^ Its inclusion failed to effectively deter use, as MDMA was prohibited before gaining popularity.

Attaining Class A, Schedule 1 controlled drug status in 1977 did not prevent MDMA’s subsequent rise as a highly popular dance drug. Approximately a decade after prohibition, MDMA emerged as the substance of choice in the acid house, rave, and club scenes of the “decade of dance” from 1988 to 1998.^9^ Initially congregating in abandoned warehouses and isolated fields, the UK rave scene transitioned to indoor, licensed premises in the early 1990s—accelerated by the 1994 Criminal Justice and Public Order Act. From the mid-1990s onwards, more legal clubs and “super clubs” opened, with MDMA remaining the preferred drug of choice for partygoers. In a landmark study from the late 1990s, Measham et al. (2001)^10^ discovered that 78% of clubbers identified ecstasy as their favorite drug for clubbing. More than two decades later, the 2019 English Festival Study revealed that among festival-goers who had ever tried MDMA, 28% still ranked it as their preferred substance—a higher percentage than for any other legal or illegal drug.^11^

While the electronic music scene embraced MDMA with gusto, studies toward medical and therapeutic use suffered greatly due to the strict scheduling laws.^12^ One critical misstep occurred in^13^ study, which erroneously used the highly addictive and more neurotoxic methamphetamine (“crystal meth”) instead of MDMA, leading to the inaccurate claim that MDMA caused severe dopaminergic neurotoxicity in primates.^13^ Though rightfully retracted, this paper has left a lingering impact on attitudes regarding MDMA’s safety in clinical settings. Applications for proposed MDMA studies were initially refused following its publication, contributing to the investigations that ultimately exposed the substance mix-up.^14^

Despite the retraction, critics continue citing this study when questioning MDMA’s viability as a therapeutic agent. However, its invalidation due to the unintended substitution of methamphetamine—a proven neurotoxin demonstrates the need for rigorous oversight in psychopharmacological research interpreting such findings. Overcoming this, and other tainted perceptions remains an obstacle for MDMA’s progression into regulated medical use.

While the mistaken use of methamphetamine in Ricaurte’s flawed study stalled clinical MDMA research for years due to perceived safety concerns, the untimely death of British teenager Leah Betts in 1996 had a similarly chilling effect on public attitudes. Betts’ death from water intoxication-induced hyponatremia^15^ was reported as direct MDMA toxicity by the tabloid media. Like the Ricaurte study’s eventual retraction, coroner’s revelations that Betts likely fell victim to misinformation about consuming water while under the influence of ecstasy, leading her to take far too much, but this could not undo the reputational damage already inflicted on MDMA. Both incidents highlight how unsupported claims can severely undermine progress in understanding drugs like MDMA.^16^

## Statistics and dynamics: the evolution of a modern entactogen

Drug use wanes and rises over time, depending on many factors including availability, purity, user apathy, and when new, more popular compounds become available. MDMA is no different. Significant changes in its use and perception across different regions and demographics have been observed, with peak-interest as a recreational drug in western countries occurring during the rave era of the 1990s and a greater focus on it’s potential as a medicine emerging in the new millennium.

Globally, the use of MDMA has remained relatively stable over the past decade. In 2016, the United Nations Office on Drugs and Crime (UNODC) reported that around 20 million people globally use MDMA annually.^17^ The 2022 World Drug Report notes that the prevalence of MDMA use remains at about 0.4% of the global population aged 15–64, steady at approximately 20 million people.^18^

The global landscape of MDMA use does however show significant regional variations, though the lower and upper estimates in the data can vary dramatically in developing countries.

Based on data from the 2022 World Drug Report, Table 1 (above) shows some insight into the trends of global MDMA use, compared with that of the UK over time.^18^ The data from The Crime Survey for England and Wales (CSEW) highlights the dynamic nature of MDMA’s popularity, with a noticeable peak in the early 2000s followed by a significant decline by 2008, only to witness a resurgence in recent years (ONS 2023). The increase from 1.6% in 1996 to 3.5% in 2001 mirrors the rave culture’s zenith, however, the subsequent drop to 1.4% by 2008 reflects both changing social attitudes and perhaps the impact of public health campaigns and tighter regulations. The increase in use by 2019, particularly among young adults aged 16–24, suggests a re-emergence of the drug as festivals, electronic music, and large-scale social events become more prevalent in mainstream youth culture.

**Table 1.**
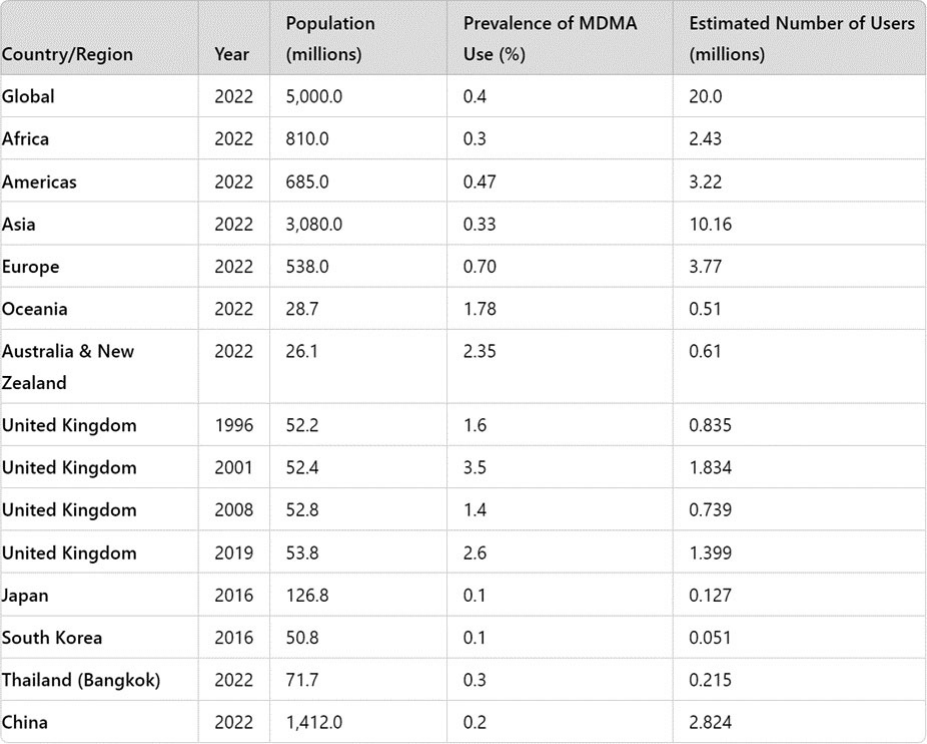
Estimated MDMA Prevalence and User Numbers Across Global Regions and Selected Countries (1996–2022).

In Asian countries like Japan and South Korea, MDMA use remains low due to strict drug laws and cultural stigmatization. However, in these countries, MDMA use appears to be on the rise, especially in cities like Seoul, Tokyo, and Osaka where the underground club scene and the influence of Western culture play significant roles in changing behavior.^19^

Southeast Asia, particularly Thailand, has also seen rising MDMA use. Bangkok stands out as a major hub where MDMA is widely available and used. The thriving nightlife and tourist influx in Bangkok likely contribute to this trend, especially as the country adopts a more liberal approach to cannabis and psychedelic-assisted therapy.^18^ MDMA use in China has seen a marked increase, especially in urban areas like Beijing and Shanghai, where it has gained popularity among young adults in the clubbing scene. This shift may be influenced by the country’s gradual adoption of Western cultural behaviors, particularly among its youth. However, the spread of MDMA use in other regions, such as Africa and the Middle East, has been slower, possibly due to cultural differences, limited substance availability, and the region’s less extensive exposure to Western drug habits.

## Pharmacology of MDMA

In the early years of MDMA production, the key ingredient as a precursor was safrole oil, derived from the roots and bark of the sassafras tree. The journey from sassafras to MDMA involves a series of chemical processes, but it all begins in the dense South-east Asian forests where these trees grow.

First, the roots and bark of the sassafras tree are harvested and then subjected to steam distillation, which separates the essential oil from the plant material. This oil is rich in safrole, which is then isomerized to isosafrole, which undergoes oxidation to form the intermediate compound MDP2P (3,4-methylenedioxyphenyl-2-propanone). Finally, MDP2P is reductively aminated to produce MDMA.

Despite an initial ban on MDMA, its use persisted, prompting the UN in the late 1990s to shift tactics by targeting the production of its key precursor, safrole, derived from sassafras oil. In 1999, the UN and European regulators banned the unlicensed production, distribution, and sale of safrole, hoping to stifle MDMA production. However, this strategy only saw a significant impact in 2008, following a massive seizure of 50 tons of sassafras oil in Thailand. This event, representing half the world’s annual supply for MDMA production, led the UN to believe they had significantly curbed MDMA availability. Yet, as safrole became scarce, underground chemists adapted by using anethole, a widely available ingredient in cosmetics. This shift inadvertently led to the production of PMA and PMMA, compounds chemically similar to MDMA but with dangerous differences, which were subsequently sold as ecstasy.^20^

In June 2008, the Cambodian government, in collaboration with the Australian Federal Police (AFP), orchestrated a dramatic media event. They incinerated 1278 drums of safrole-rich oil. This quantity of oil could have yielded an estimated 245 million ecstasy tablets, with a street value of $7.6 billion in Australia, according to the AFP. As thick black plumes of smoke billowed into the sky, Australian police officers, clad in chemical suits and breathing apparatus, stood by, overseeing the public destruction.

The safrole crackdown had a twofold purpose—to curb the production of MDMA and protect the threatened sassafras trees from extinction. The aggressive eradication efforts, however, resulted in a significant shortage of safrole oil, significantly impacting the availability of MDMA on the streets. This shortage drove chemists to seek alternative routes and precursors for MDMA synthesis, some of which were less efficient or more dangerous. It was during this period of drought that mephedrone, a then-legal synthetic cathinone became popular and filled the vacuum that MDMA left in the clubs and festivals of the UK.^21^ Attempts to make compounds that had similar effects and were sold as MDMA through using an alternative natural oil led to the sale of related but more toxic amphetamines, PMA (paramethoxyamphetamine) and PMMA (paramethoxymethamphetamine)—substances that can cause several severe adverse effects due to strong stimulant properties and narrow therapeutic window.

PMA itself is a potent and toxic compound, and the above risks are compounded when taken inadvertently in a recreational environment, mainly due to redosing (as the effects are delayed compared to MDMA), and the risks of overheating. These incidents are but one of the many examples of banning a drug leading to more harmful alternatives. To quote,^22^ “These laws and conventions are not evidence-based, they are based on morality and politics and are therefore failing at their legal duties.”

At its molecular core, MDMA is a ring-substituted phenethylamine, bearing a structural similarity to methamphetamine and the hallucinogen mescaline.^23^

MDMA possesses a chiral center, giving rise to a pair of mirror-image enantiomers—a racemic mixture of which is typically synthesized. The S (+) isomer is responsible for MDMA’s psychostimulant and empathogenic properties, while its mirrored form, the R (−) isomer, is attributed to the hallucinogenic effects. These latter effects are reportedly much milder than that of classic psychedelics such as LSD, but greater than that of amphetamine.^24^ The most common effects include feelings of euphoria, happiness, stimulation, increased energy levels, extroversion, closeness to others, heightened empathy, sociability, improved mood, mild sensory distortions, and altered perception of colors and sounds.^23^ These sensory shifts, coupled with emotional bonding, can enhance the experience of music (especially when this music comprises repetitive beats in a collective environment), as well as a heightened level of engagement within a psychotherapeutic setting.

## Effects on neurotransmitters

At the heart of MDMA’s mechanisms of action lies the release and reuptake inhibition of serotonin (5-HT), dopamine, and norepinephrine.^25^ The serotonergic effects of MDMA are mediated primarily through the reversal of the serotonin transporter (SERT), resulting in a potent release of 5-HT into the synaptic cleft.^26^ This surge of serotonin is thought to underlie the subjective experiences of empathy, emotional openness, and interpersonal closeness associated with MDMA.^27^ Additionally, MDMA’s affinity for various 5-HT receptor subtypes, particularly 5-HT2A and 5-HT2C, may contribute to its psychoactive properties.^28^

MDMA also affects dopamine, facilitating its release of dopamine and inhibiting its reuptake.^29^ This dopaminergic modulation is implicated in the feelings of euphoria, increased energy, and enhanced sensory perception observed with MDMA use.^29^ Furthermore, the noradrenergic effects of MDMA, mediated through the release and reuptake inhibition of noradrenaline may contribute to its subjective effects, including increased heart rate and blood pressure.^30^

Table 2 illustrates the comparative effects of MDMA on neurotransmitter systems, showing a pronounced impact on serotonin, with moderate effects on noradrenaline and dopamine.MDMA is readily absorbed following oral administration, with peak plasma concentrations typically occurring within 1–2 h.^23^ It is then extensively metabolized by the liver, primarily via O-demethylenation and N-dealkylation pathways, resulting in the formation of several metabolites. The body excretes the unchanged parent compound as well as 3,4-methylenedioxyamphetamine (MDA), the free and conjugated forms (glucuronidated/sulfated) of 4-hydroxy-3-methoxymethamphetamine (HMMA) and 4-hydroxy-3-methoxyamphetamine (HMA^31^).

**Table 2.** The comparative effects of MDMA on three neurotransmitter systems, demonstrate its pronounced impact on serotonin, with moderate effects on noradrenaline and dopamine

System	Mechanism of Action	Activity Level
Noradrenaline	MDMA increases the release of noradrenaline (NA) from presynaptic neurons, leading to heightened alertness and energy.	Moderate
Serotonin	MDMA causes a significant release of serotonin (5-HT) from presynaptic neurons and inhibits its reuptake, resulting in enhanced mood and emotional connectivity.	High
Dopamine	MDMA induces the release of dopamine (DA) and blocks its reuptake, contributing to increased feelings of pleasure and reward.	Low to Moderate

**Table 3.** Outlines key clinical trials investigating MDMA-assisted psychotherapy for both PTSD and AUD, including the pivotal Bristol-Imperial-MDMA-for-Alcoholism (BIMA) trial. These studies span various phases of research, with participant numbers ranging from small pilot trials to large-scale Phase 3 studies. Notably, MAPS’ Phase 3 trials enrolled over 100 participants and demonstrated the efficacy of MDMA-assisted therapy in significantly reducing PTSD symptoms

Study	Phase	Condition	Participants	Key findings
Mithoefer et al. (2018)^37^	Phase 2	PTSD	26 total 7 received 30 mg MDMA plus psychotherapy 7 received 75 mg MDMA plus psychotherapy 12 received 125 mg MDMA plus psychotherapy.	MDMA-assisted psychotherapy significantly reduced PTSD symptoms, with 68% of participants no longer meeting PTSD criteria 2 mo after treatment.
Ot’alora et al.^36^	Phase 2	PTSD	28 total 6 received 40 mg MDMA plus psychotherapy 9 received 100 mg MDMA plus psychotherapy 13 received 125 mg MDMA plus psychotherapy.	Demonstrated the safety and efficacy of MDMA-assisted psychotherapy for severe, treatment-resistant PTSD, with 76% of participants experiencing significant improvement in symptoms.
BIMA Trial^38^	Safety and tolerability study	Alcohol use disorder (AUD)	14 total	MDMA-assisted psychotherapy for AUD showed that participants reduced alcohol consumption from an average of 130.6 units per week to 18.7 units per week at 9-mo follow-up. The treatment was well tolerated, with no unexpected adverse events.
MAPS Phase 3 Trial 1 (Mitchell et al., 2021)^39^	Phase 3	PTSD	90 total 79 completed at the study endpoint (18 wk) 42 received MDMA 37 received placebo	MDMA-assisted psychotherapy showed significant reductions in PTSD symptoms compared to placebo, with 67% of participants no longer meeting PTSD criteria after treatment.
MAPS Phase 3 Trial 2^40^	Phase 3	PTSD	104 total 94 completed at the study endpoint (18 wk) 51 received MDMA 43 received placebo	Confirmed the efficacy of MDMA-assisted therapy in reducing PTSD symptoms, with 71.2% of MDMA-treated participants experiencing clinically significant improvement.

**Figure 1.**
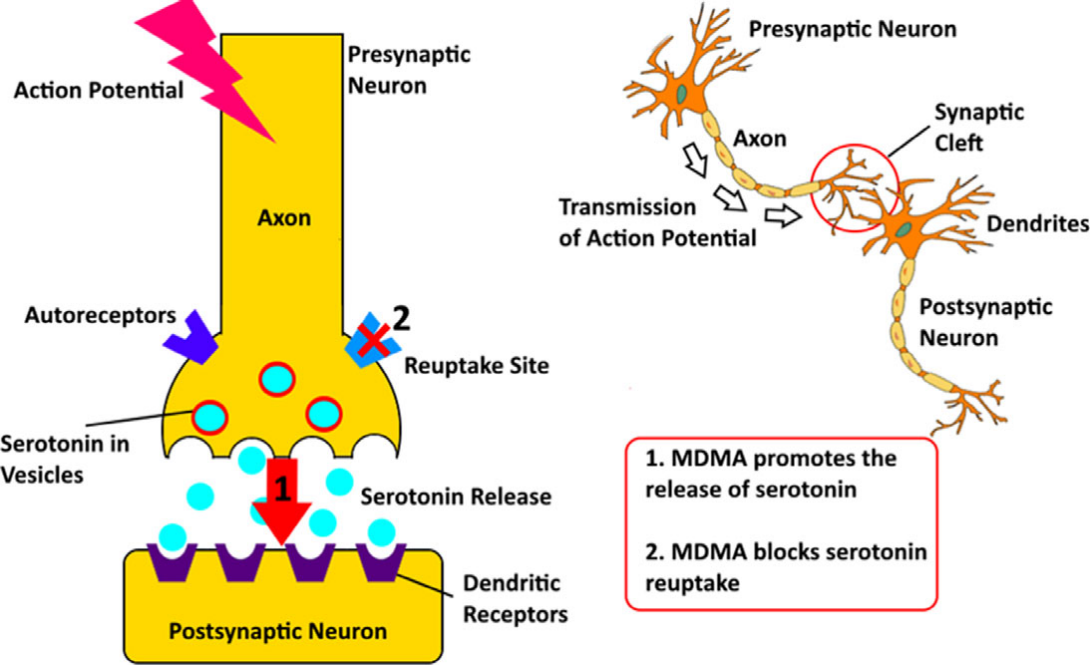
Mechanism of MDMA’s effects on serotonin neurotransmission. MDMA promotes serotonin release from presynaptic neurons while simultaneously blocking its reuptake, leading to increased serotonin levels in the synaptic cleft. .

Figure 1 demonstrates the interaction MDMA has in interrupting serotonin release and reuptake in the brain. Under normal circumstances, serotonin is stored in vesicles within the presynaptic neuron and released into the synaptic cleft in response to an action potential. Once released, serotonin binds to receptors on the postsynaptic neuron, transmitting the signal and then being reabsorbed into the presynaptic neuron through reuptake sites to terminate the signal.^32^ MDMA disrupts this cycle in two significant ways. First, MDMA promotes the release of serotonin from the presynaptic neuron into the synaptic cleft. It achieves this by entering the presynaptic neuron through the serotonin transporter and inducing the release of serotonin stored in vesicles. This massive release floods the synaptic cleft with serotonin, leading to increased stimulation of the postsynaptic receptors.^33^ Second, MDMA blocks the reuptake of serotonin by binding to and inhibiting the serotonin transporter. This action prevents serotonin from being reabsorbed back into the presynaptic neuron, prolonging its presence and activity in the synaptic cleft.

Carhart-Harris et al.,^34^ investigated the impact of MDMA on both the subjective experience and brain activity related to personal memories, to help understand of mechanisms of the therapeutic efficacy of MDMA, particularly in PTSD. In this study, participants were given MDMA and then asked to recall their most positive and negative autobiographical memories. The researchers used blood oxygenation level-dependent functional magnetic resonance imaging (BOLD-fMRI) to measure brain activity during these recall sessions. They found that MDMA significantly altered the emotional intensity of these memories. Participants reported a reduction in the negative emotional intensity associated with their worst memories and an enhancement of the positive feelings linked to their best memories. The BOLD-fMRI data indicated that these subjective changes were accompanied by alterations in brain regions involved in emotion and memory processing, such as the amygdala and hippocampus.^34^

This finding is crucial as it suggests that MDMA may help individuals reprocess traumatic memories by reducing their negative emotional impact allowing patients to extinguish them.^35^

## Psychotherapeutic approaches with MDMA

**Figure 2.**
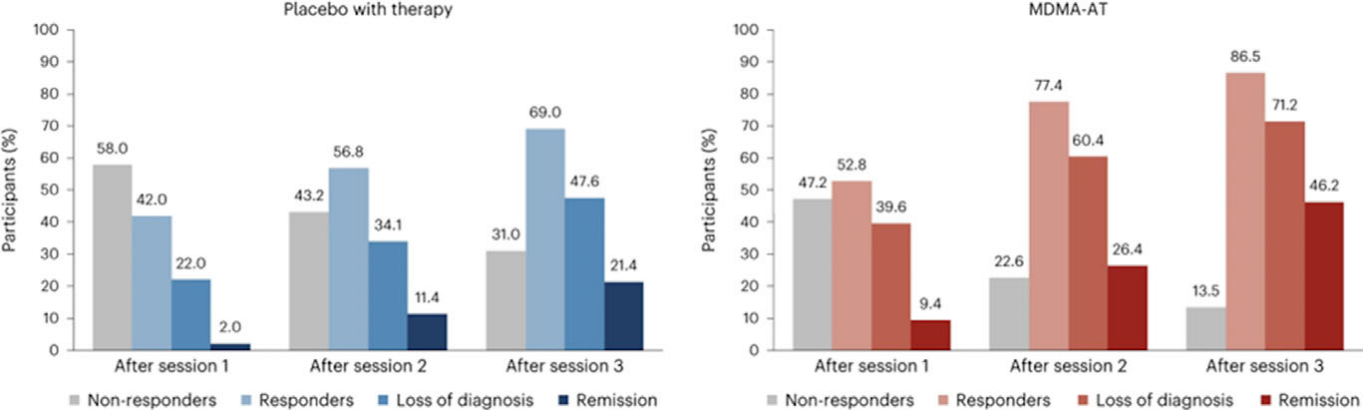
A comparison between the effects of therapy combined with placebo versus MDMA-AT across three treatment sessions. The bars represent the percentage of participants categorized into four groups: nonresponders, responders, those with a loss of diagnosis, and those in remission. Notably, MDMA-AT shows a significant increase in the proportion of participants achieving remission and loss of diagnosis after each session, particularly after the third session, compared to the placebo group.^36^

MDMA’s therapeutic potential has garnered significant attention in recent years, primarily driven by groundbreaking research into MDMA-assisted psychotherapy (MDMA-AT) for posttraumatic stress disorder (PTSD). “The Multidisciplinary Association for Psychedelic Studies (MAPS) initiated Phase 3 clinical trials investigating the efficacy of MDMA-assisted therapy (MDMA-AT) for treatment-resistant PTSD. These trials are now overseen by Lykos Therapeutics, formerly known as MAPS Public Benefit Corporation (MAPS PBC), which is driving the regulatory and commercial pathways for these therapies.^36^ The results have been remarkably encouraging, demonstrating significant and sustained reductions in PTSD symptom severity compared to traditional psychotherapy alone (see Figure 2).

There are certain time windows during brain development when the nervous system is in a highly plastic state. During these periods, the brain’s adaptive capacity is highly sensitive to specific environmental inputs. These “critical periods” allow for greater neuronal and biochemical growth early in life, which then becomes more constrained as the brain matures. In disorders, the closing of these critical periods limits the brain’s ability to adapt, even if optimal conditions are restored later. Finding ways to reopen critical periods has been an important goal for translational neuroscience research.

In a 2019 study by Nardou et al., evidence that regulation of a specific form of synaptic plasticity (long-term depression) mediated by the hormone oxytocin in the nucleus accumbens region of the brain establishes a critical period for social reward learning.^41^ Remarkably, a single dose of the drug MDMA was able to reopen this critical period by increasing oxytocin-dependent long-term depression. The reopening required activating oxytocin receptors in the nucleus accumbens and could be mimicked by directly stimulating oxytocin inputs to this region.

These findings shed light on the potential origins of neurodevelopmental disorders involving social impairments, as well as other conditions influenced by social factors or social trauma. Understanding how to reopen critical periods could therefore lead to new therapeutic strategies.

MDMA’s therapeutic potential finds expression in its ability to facilitate exposure therapy, a cornerstone of PTSD treatment. The sudden, involuntary resurgence of emotions experienced during the initial traumatic event is a key aspect of PTSD’s symptomatology. Nightmares, flashbacks, and triggering sensory input such as sounds and images all contribute to the ongoing trauma of suffering with PTSD.^42^ Consequently, contemporary treatments aim to reactivate and subsequently extinguish these negative emotional memories. In healthy volunteers, MDMA has demonstrated a capacity to attenuate the impact of negative memories, whether autobiographical or induced through controlled exposure to aversive stimuli.^34^

**Figure 3.**
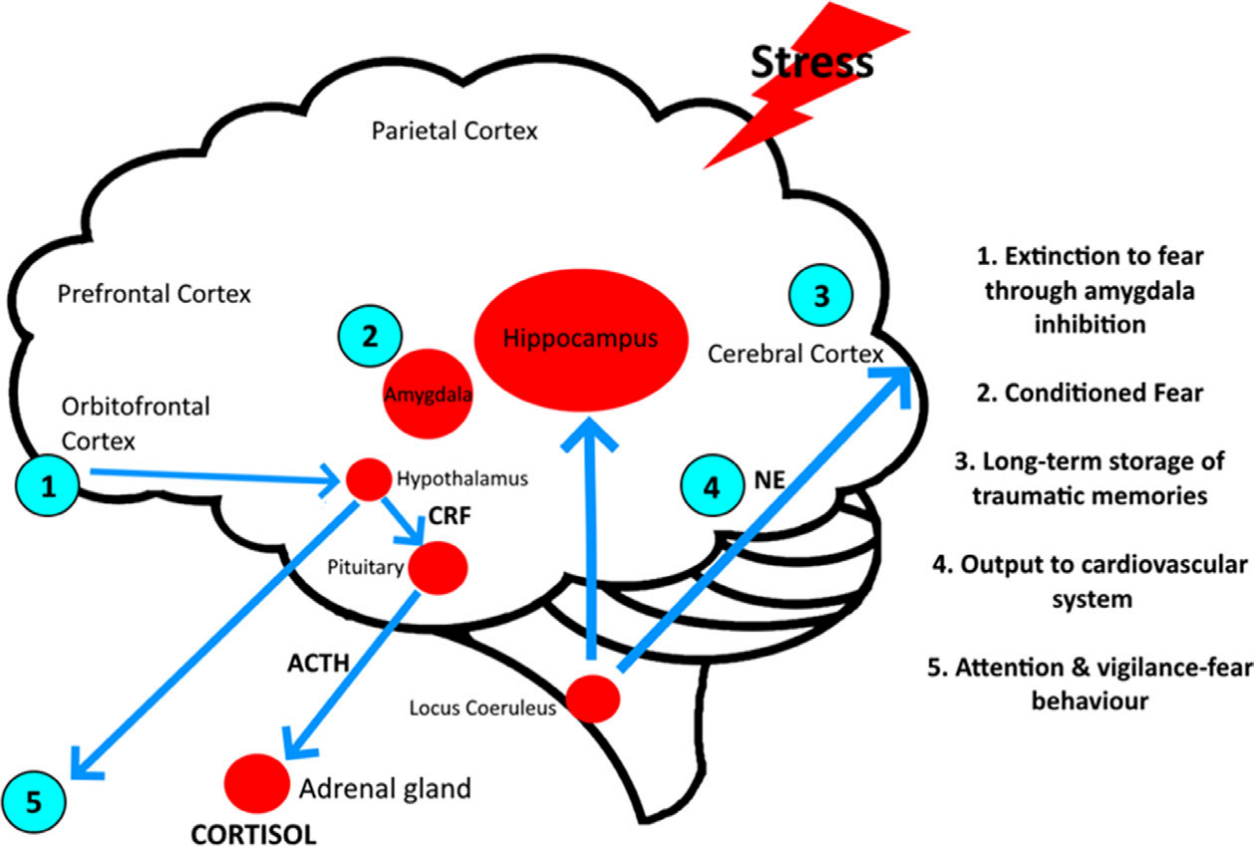
The effects of stress on the brain and the impact on subsequent hormonal change and behavior.

Stress is a complex physiological response that affects various regions of the brain through the interaction between signals, regions, hormones, and neurotransmitters. Figure 3 illustrates the pathways and interactions involved in the brain’s response to stress. Understanding how MDMA interacts with the brain’s stress pathways can assist in developing therapeutic approaches toward reducing stress and anxiety.

One of the primary ways MDMA exerts its effect is through the enhancement of the prefrontal cortex (PFC) function. The PFC is crucial in regulating emotions and executive functions, but its ability to inhibit the amygdala is compromised under chronic stress. The amygdala, responsible for processing fear and anxiety, becomes hyperactive under stress. MDMA significantly reduces amygdala activity, leading to decreased fear and anxiety responses. MDMA enhances connectivity between the PFC and the amygdala, improving the PFC’s capacity to regulate emotional responses (see Figure 3). This improvement is likely mediated by the increased release of serotonin (5-HT) in the PFC, resulting in enhanced mood—a key aspect of the appeal of MDMA in both therapeutic and recreational situations.^43^

Modulation of emotional processing and reduction of the conditioned fear response becomes difficult in psychotherapy when revisiting traumatic experiences. MDMA, by calming this effect, enhances the efficacy of psychotherapy when used in carefully controlled environments.^36^

The hippocampus, essential for memory formation and emotional regulation, suffers under chronic stress, often leading to memory impairment and emotional dysregulation. MDMA promotes neurogenesis in the hippocampus, enhancing its ability to process and store memories. By increasing brain-derived neurotrophic factor (BDNF) levels, MDMA supports neuronal growth and resilience, counteracting the detrimental effects of cortisol on hippocampal neurons.^44^

Cortisol prepares the body for a “fight or flight” response by increasing blood sugar levels and suppressing nonessential functions. Chronically elevated levels of cortisol can lead to various health issues, including immune suppression and metabolic disturbances.^45^

## Neurological factors and stress response

The feed-forward loop between noradrenergic/CRF-containing neurons, the locus coeruleus, and the paraventricular nucleus (PVN) is a crucial mechanism in the stress response. This loop involves reciprocal projections where CRF-releasing neurons in the PVN activate the locus coeruleus, enhancing the release of noradrenaline. In turn, noradrenaline feedback to the PVN amplifies CRF release, creating a self-reinforcing cycle that heightens HPA axis activation and stress hormone secretion.^46^ This loop is particularly relevant in conditions like PTSD, where hyperactivation can perpetuate chronic stress responses. MDMA’s therapeutic effects may partly arise from its ability to disrupt this loop by attenuating overactivity in both the locus coeruleus and CRF-releasing neurons, thereby normalizing the HPA axis.

The hypothalamus–pituitary–adrenal (HPA) axis governs the stress response through the release of cortisol from the adrenal glands. Stress triggers the release of corticotropin-releasing factor (CRF) from the paraventricular nucleus (PV) of the hypothalamus, which activates the pituitary gland to secrete adrenocorticotropic hormone (ACTH). ACTH then stimulates the adrenal glands to release cortisol, completing the HPA axis activation.

MDMA modulates the activity of this axis by affecting several key components. It reduces the release of CRF from the hypothalamus and subsequently lowers ACTH secretion from the pituitary gland, leading to decreased cortisol production. Furthermore, MDMA attenuates the hyperactivity of the locus coeruleus—a region that plays a significant role in the stress response—by reducing the release of noradrenaline (norepinephrine). This dampening effect helps normalize the HPA axis and mitigate the heightened stress response typically observed in PTSD.^47^^,^ ^48^

The dual action of MDMA-instigated serotonin and oxytocin release may help mitigate the adverse effects of stress and enhance emotional resilience.^49^ The interruption of stress pathways by MDMA is likely a key factor in its value for treating PTSD patients with MDMA-AT. By dampening the hyperactive stress response and enhancing emotional regulation, MDMA-AT shows promise in alleviating PTSD symptoms. MDMA’s ability to promote neurogenesis and enhance neuroplasticity in the hippocampus and other brain regions suggests long-term benefits in restoring normal brain function and resilience to stress.^34^

## Safety and tolerability of MDMA

While there have been many studies into the beneficial uses of MDMA in psychotherapy, it is important to be considerate of the dangers that prolonged, or even short-term MDMA exposure can involve.^1^

In controlled clinical settings, MDMA has generally been well-tolerated, with a favorable safety profile when administered under medical supervision and appropriate screening protocols.^50^ However, some adverse effects have been reported, with their severity and frequency varying across studies.

Common acute adverse effects include increased blood pressure, heart rate, body temperature, and mydriasis (pupil dilation^51^) during drug administration. These physiological changes necessitate careful monitoring and management, particularly in individuals with preexisting cardiovascular or thermoregulatory conditions. Additionally, psychological effects such as anxiety and jaw-clenching (gurning) are common during treatment, with headache, muscle tension, dizziness, fatigue, and low mood also reported by some participants.^36^

Long-term effects and considerations for repeated dosing are also subject to ongoing investigation. Concerns have been raised regarding potential neurotoxicity, particularly in the serotonergic system, although the clinical relevance and reversibility of these findings remain subjects of debate.^52^ Cognitive impairments, including deficits in memory and attention, have been reported in some studies,^53^ although the evidence is inconsistent and influenced by various confounding factors such as polydrug use, focus on recreational users, and impact of duration of use or dosing.^54^

To mitigate potential risks, clinical protocols for MDMA-AT involve comprehensive medical and psychological screening, careful dose selection, and rigorous monitoring during and after administration.^50^ Additionally, researchers have explored the use of adjunctive medications and interventions to manage specific adverse effects, such as the administration of benzodiazepines for anxiety, should the experience become too intense or overwhelming.^4^^,^ ^55^

**Figure 4.**
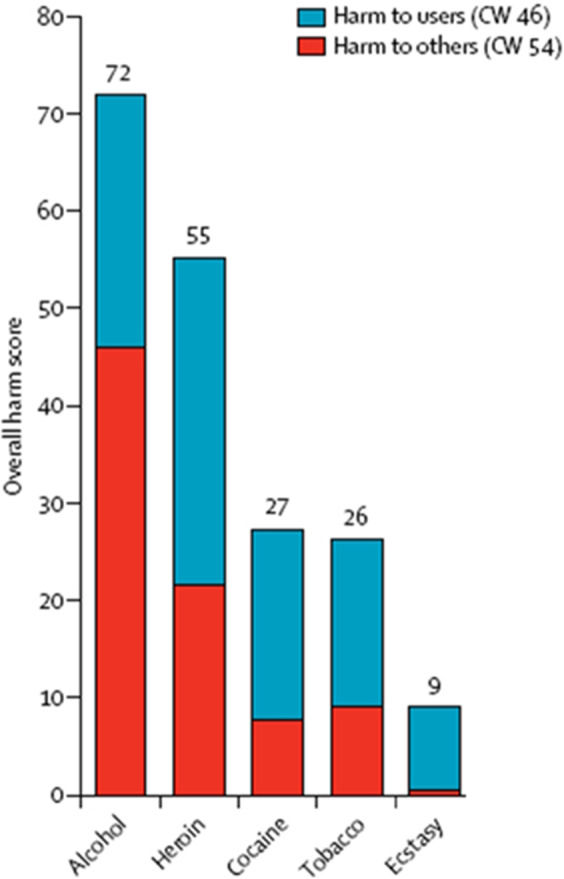
Overall harm scores for various substances are divided into two components: harm to users and harm to others. The harm caused directly to individuals who use the substance includes factors like addiction, overdose, and physical or mental health problems. Harm to others may include impacts like crime, accidents, and familial or social factors.^57^

While the overall safety profile of MDMA in controlled clinical settings is favorable, ongoing research and vigilance are necessary to fully understand its long-term effects and potential risks. As the field progresses, the development of standardized protocols and guidelines for MDMA administration and risk mitigation will be crucial for ensuring the safe and responsible integration of MDMA into clinical practice. In a 2007 study, Professor David Nutt explored the comparative harms of different substances, controversially concluding that alcohol and tobacco were more harmful than many illegal drugs, including MDMA.^56^ This study played a crucial role in shifting public and academic perceptions about the relative dangers of these substances (see Figure 4).

To further support the findings by Nutt et al., several other studies across different regions arrived at similar conclusions regarding the relative harms of substances like alcohol and tobacco versus illegal drugs such as MDMA. Van Amsterdam et al. (2015) in Europe, Bonomo et al.^58^ in Australia, and Crossin et al.^59^ in New Zealand, all conducted research that reinforced the notion that alcohol and tobacco present more societal harm compared to MDMA, further shifting both public and academic perspectives on drug policy. Nutt’s work has also delved into the therapeutic potential of MDMA-AT, among other psychedelic-assisted therapies. His research has shown that MDMA can help reduce the fear response in patients, making it easier for them to engage in therapeutic processes and confront traumatic memories.^60^ These findings have been pivotal in supporting the ongoing clinical trials investigating MDMA as a treatment for multiple behavioral disorders, that all seem to have a common compounding factor as the root cause: trauma, especially in childhood.

### Treatment protocols

Across MDMA-AT studies these tend to be relatively similar, based on the work undertaken by MAPS, among other institutions. The most recent protocol from the phase 3 study is detailed below.^36^

#### Preparation Phase

Patients undergo three 90-min preparatory sessions with trained therapists. These sessions are designed to build trust, provide information about MDMA and the therapy process, and establish a therapeutic alliance. The goal is to prepare patients mentally and emotionally for the MDMA-assisted sessions.

#### MDMA-assisted therapy sessions

Patients participate in 3 d-long MDMA-AT sessions, each spaced about a month apart. During these sessions, patients are administered a dose of MDMA (80–120 mg) in a controlled clinical setting, with the option of a supplemental dose (40–60 mg) if needed. These sessions last approximately 8 h and are conducted by a pair of therapists, typically one male and one female, to provide balanced support. The therapists use a nondirective approach, allowing patients to process their experiences in a supportive environment.

#### Integration phase

Following each MDMA session, patients engage in three 90-min integration sessions with their therapists. These sessions are crucial for helping patients make sense of their experiences during the MDMA sessions, integrating insights into their daily lives, and addressing any arising issues. The integration phase ensures that the therapeutic benefits are consolidated and sustained over time.

The study emphasizes the importance of set (patient’s mindset), setting (physical and social environment), and the supportive presence of therapists throughout the process. It exemplifies the necessity of rigorous screening and monitoring to manage potential risks, including psychological distress or adverse reactions to MDMA.

**Figure 5.**
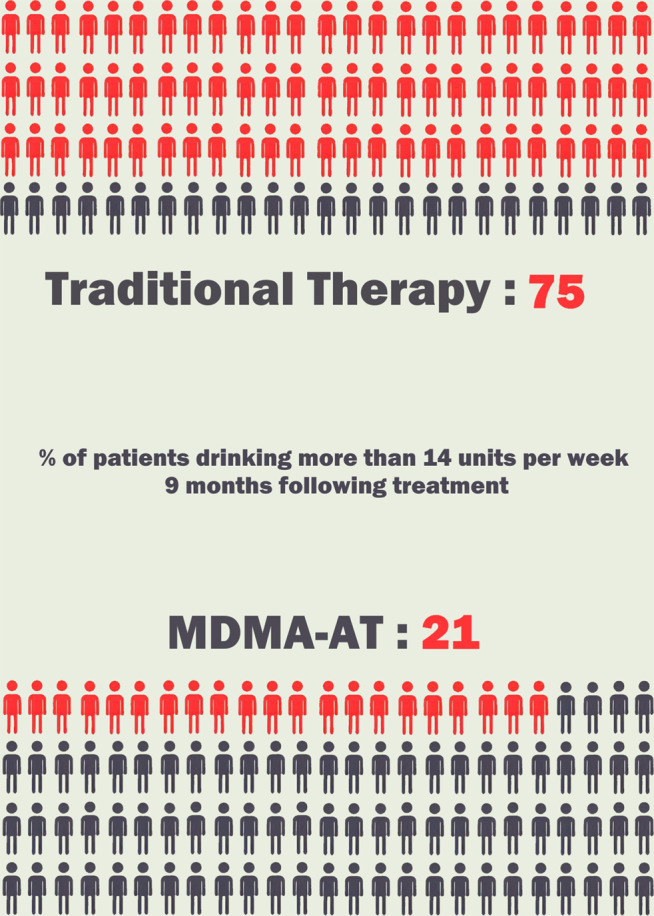
Results of the first study of safety and tolerability of MDMA-AT in patients with alcohol use disorder at 9 month follow-up, showing % of patients using more than 14 units of alcohol per week.^38^

The BIMA study focused on the outcomes of MDMA-AT for alcohol use disorder (AUD); the first study of its kind, considering a leading focus for MDMA-AT has often been on other disorders rooted around trauma such as PTSD rather than addiction. This study showed primarily that MDMA-AT when used as a treatment for AUD was safe and well-tolerated, but also potentially effective in reducing alcohol consumption and maintaining abstinence over an extended period.^38^ The study had a relatively small sample size and was an open-label, nonplacebo-controlled study. Therefore, all patients knew they would be receiving MDMA, which could potentially influence the outcomes. However, only 21% of participants were drinking in excess of 14 units of alcohol per week at the 9-month follow-up. This is a significant reduction compared to the 75% observed in the Outcomes Study, a prior study using a similar number of participants in the same geographical area, treated with psychotherapy alone (see Figure 5).

Comorbid disorders such as depression, dissociative disorders, and substance use disorders are frequently observed in patients with PTSD. Despite their high prevalence in trauma-affected populations, these conditions are often excluded from clinical trials involving MDMA-AT. Comorbidities complicate treatment and currently lack FDA-, EMA-, or MHRA-approved therapies that address both PTSD and co-occurring disorders. MDMA-AT shows promise in treating PTSD with comorbidities, offering a potential solution where no approved medications are currently available.

### MDMA and acute trauma events

Real-world evidence for MDMA as a protective agent against PTSD came from an unexpected event: the October 7 attacks by Hamas in Israel in 2023, which resulted in nearly 1500 civilian deaths. This event, marked by its profound psychological impact on the affected population, provides a crucial case study for understanding the efficacy and dynamics of MDMA in a real-world setting of acute trauma.^61^

The Supernova festival attack allowed researchers to investigate the effects of psychoactive substances on trauma processing during a mass trauma event. Survivors who consumed MDMA during the trauma showed enhanced coping abilities and improved clinical outcomes, including better sleep and social support, compared to those who consumed alcohol, cannabis, or no substances. The energizing and prosocial effects of MDMA may have provided survivors with immediate benefits, reducing fear and aiding escape. In the long term, MDMA was associated with better trauma processing during the peritraumatic period, leading to fewer PTSD symptoms. This study offers novel insights into how MDMA might protect against the psychological impact of severe trauma, although it acknowledges natural experiment limitations, including survivor bias and the inability to control other influencing factors. Future longitudinal research will further explore the cognitive and physiological mechanisms underlying these protective effects.^61^

### Ethical and regulatory considerations

Navigating the world of MDMA-AT means understanding not only its effects and potential benefits but also the ethical and regulatory challenges that come with its use which presents many challenges and complexities. The imperatives of patient safety and scientific rigor intersect with societal norms and legal frameworks, driving some patient-focused therapists to perform sessions in unapproved environments such as retreats or private psychotherapy practices. Studies and reports indicate that the underground network of psychedelic therapists is particularly active in regions where there is a higher prevalence of psychedelic research and advocacy, such as the United States, Europe, and Canada. In the US alone, estimates from interviews and community reports suggest there could be 100 of underground therapists offering such services, but these numbers are speculative and based on informal networks and practitioner testimonials.

One of the main concerns is the question of autonomy and informed consent, as individuals grappling with mental illness, addiction, or behavioral disorders struggle with the delicate balance between vulnerability and agency.^62^ In the context of MDMA-AT, where altered states of consciousness and heightened emotional vulnerability may blur the boundaries of consent, ensuring the ethical conduct of research and practice is paramount.

From the stigma surrounding mental illness to the taboo of psychedelic substances, societal attitudes play a pivotal role in shaping the ethical contours of MDMA’s medical use.^63^ As advocates and researchers strive to destigmatize mental illness and elevate the discourse surrounding psychedelic therapies, there are very few countries that have taken the leap to allow legal access to MDMA-AT outside of clinical trials, the exception being Australia. MDMA was originally classified as a Schedule 9 substance under the Australian Therapeutic Goods Administration (TGA) classification system (equivalent to schedule 1 in the USA and UK), claiming it was a dangerous substance with a high potential for abuse and no accepted medical use. This classification effectively prohibited its use outside of strictly controlled research settings. The initial legal framework was shaped by international drug control treaties and domestic legislation, reflecting a global stance on the war against drugs. The shift towards considering MDMA for therapeutic use began with accumulating scientific evidence demonstrating its efficacy in treating PTSD. Studies, including those conducted by the MAPS, gained significant advocacy from researchers, healthcare professionals, and patient groups, thus providing a crucial shift in perspective and pressure on the government and legislative bodies. They argued that the potential benefits of MDMA-AT warranted a re-evaluation of its legal status.

One of the significant regulatory challenges was the need to reclassify MDMA as a Schedule 8 substance, which is designated for controlled drugs that require a prescription. This reclassification involved several steps:

- **Review of scientific evidence:** The TGA conducted a thorough review of the available scientific literature on MDMA’s safety and efficacy. This review was crucial in building a case for its medical use.
- **Clinical trial regulations:** Establishing a regulatory framework for clinical trials involving MDMA was another critical step. This included developing guidelines for safe administration, monitoring, and ethical conduct in trials to ensure patient safety.
- **Public and expert consultations:** The TGA engaged in consultations with medical experts, researchers, and the public. These consultations provided a platform for discussing the potential risks and benefits of MDMA-AT and addressed concerns related to abuse and dependency.
- **Post-market surveillance:** The authorities also planned for robust postmarket surveillance to monitor the outcomes of MDMA therapy in real-world settings, ensuring any adverse effects were promptly identified and addressed. The ethical challenges associated with MDMA legalization were also multifaceted, involving concerns about patient safety, informed consent, and potential misuse. Authorities addressed these challenges through several strategies.
- **Informed consent processes:** Ensuring that patients were fully informed about the potential risks and benefits of MDMA therapy was paramount. Detailed informed consent processes were developed, requiring patients to acknowledge understanding the treatment and its experimental nature.
- **Therapist training and certification:** To mitigate risks, only trained and certified therapists were allowed to administer MDMA-assisted psychotherapy. This training included handling adverse reactions and understanding the psychological effects of MDMA.
- **Patient selection criteria:** Strict criteria were established for patient selection to ensure that only individuals who could potentially benefit from the therapy, and who did not have contraindications, were enrolled in treatment programs.
- **Ethics committees oversight:** Institutional ethics committees played a vital role in overseeing clinical trials and therapeutic applications. These committees ensured that the research and treatment adhered to ethical standards and protected patient welfare.

Another significant challenge is overcoming the public stigma associated with MDMA, largely stemming from its association with recreational drug use. Authorities and advocates undertook public education campaigns to shift the narrative, highlighting MDMA’s potential as a therapeutic tool rather than a recreational drug. Success stories from clinical trials and personal testimonies from patients who benefited from MDMA-AT were instrumental in changing public perception.

In the face of these challenges, perspectives on risk-benefit assessments and patient safety emerge as crucial considerations in the ethical and regulatory discourse surrounding MDMA-AT. Balancing the potential risks of adverse effects and long-term harm against the promise of therapeutic benefit requires a nuanced understanding of both the science and the ethics at play . As researchers and regulators grapple with these complex calculations, the imperative of patient safety remains paramount, guiding decisions and shaping the future trajectory of MDMA-AT.

### Future directions and challenges

MDMA is being put forward by several leading bodies for a potential treatment for soldiers and civilians in Ukraine following the invasion by Russia in 2022.

Ukrainian MP Dmytro Gurin’s advocacy for the use of psychedelics, particularly MDMA, to treat PTSD in combat veterans has been raised in the European Parliament.^64^ Gurin argues that Ukraine, given its high number of trauma-affected individuals due to ongoing conflict, could benefit from pioneering psychedelic therapies. He emphasizes the potential of MDMA to significantly alleviate PTSD symptoms, presenting Ukraine as an ideal test environment for these treatments.

Gurin highlights the urgency due to the high prevalence of trauma among Ukrainian soldiers, expressing the need for legal reforms to allow clinical trials and therapeutic use of psychedelics in Ukraine. The UPRA website outlines the organization’s mission to promote the legal therapeutic use and scientific research of psychedelics in Ukraine.^65^ The organization advocates for the integration of psychedelic-assisted therapy into mainstream medical practice by focusing on legislative change, training healthcare professionals, and providing patient support. By influencing the European Parliament, the organization hopes to accelerate progress toward significant modifications in existing drug laws, which could then spread across multiple countries, overcoming what has traditionally been a slow and contentious process.

Effective implementation of MDMA-AT requires training therapists in psychedelic-assisted therapy and establishing clinical infrastructure for safe administration. The path to widespread acceptance and implementation of MDMA-AT remains complex, fraught with regulatory challenges, and the need for rigorous long-term safety studies.^60^ As we move forward, it is crucial to balance the potential benefits of MDMA-AT with careful consideration of its risks and responsible use protocols. The ongoing clinical trials and evolving regulatory landscape suggest that we may be on the cusp of a paradigm shift in mental health treatment, one that could offer new hope to those struggling with treatment-resistant conditions.^66^ As research into MDMA progresses, growing evidence continues to demonstrate its promising role in shaping the future of psychiatry and addiction treatment.

MDMA has long been heralded as a groundbreaking tool in the treatment of PTSD and other behavioral disorders. Advocates, researchers, and therapists have worked tirelessly over the decades to integrate MDMA into mainstream psychotherapy, overcoming numerous legal and scientific hurdles. However, in August 2024, the trajectory of MDMA-AT faced a significant setback when the US Food and Drug Administration (FDA) advisory committee voted overwhelmingly (9–2) against approving MDMA as a therapeutic tool for PTSD.^67^

The FDA’s decision to decline MDMA for PTSD therapy was primarily based on what the advisory committee viewed as significant gaps in the research. The committee’s scrutiny extended to the methodologies and ethical considerations within the MDMA trials, which they argued fell short of the standards required for approval. Ingmar Gorman, a psychologist deeply involved in the MDMA clinical trials, expressed frustration, stating that the data presented wasn’t given the careful attention it deserved.^68^ Gorman and others in the field had hoped that the compelling evidence demonstrating MDMA’s efficacy in treating PTSD would lead to its acceptance. Yet, the committee’s vote indicated otherwise, signaling that the bar for approving new therapies, particularly those involving psychedelics, remains high.

One challenge, and subsequent factor considered by the FDA in their assessment of MDMA-AT, is the difficulty of concealing placebo treatments. Participants may easily recognize the distinct subjective effects of MDMA. This lack of an active placebo introduces the risk of unblinding and biases in outcome measures. There is however extensive work being undertaken to address this issue. Another area of concern is the perceived lack of standardized protocols and scientific rigor in MDMA-AT trials. A growing body of literature is therefore focusing on enhancing methodological rigor in psychedelic trials.^69^

Publication of clinical trial designs and outcomes offers a framework that aligns with regulatory expectations.^70^ Such efforts not only bolster the credibility of MDMA-AT research but also provide regulatory agencies with the robust data necessary for future approval. As the field advances, continued adherence to these rigorous standards will be critical in establishing MDMA-AT as a safe and effective treatment for PTSD and other conditions.

The decision by the FDA is not just a blow to MDMA research but also impacts the broader psychedelic research community. Frederick Barrett, director of the Johns Hopkins Center for Psychedelic and Consciousness Research, emphasized the need for researchers to “double down on the most rigorous methods”.^68^ This reflects a growing sentiment that, while the therapeutic potential of psychedelics like MDMA, psilocybin, and ketamine is promising, there must be an unrelenting focus on the precision, safety, and ethics of clinical trials moving forward. The decision also raised concerns regarding the influence of external interests such as ‘Big Pharma’ and alcohol industry lobbyists, who could stand to lose if MDMA were widely adopted as a treatment, further muddying the waters around this critical moment for psychedelic research.

A critical voice in this ongoing debate has been Rick Doblin, founder of the Multidisciplinary Association for Psychedelic Studies (MAPS) and a long-time advocate for MDMA-AT. In a surprising turn of events, Doblin announced his resignation from the board of Lykos Therapeutics, citing personal and professional reasons. His departure has raised questions about the future direction of MAPS and its efforts to bring MDMA-AT into mainstream practice. Many believe his resignation could slow down advocacy efforts at a crucial moment when the field needs strong leadership to navigate the regulatory and scientific challenges posed by the FDA decision.

The FDA’s decision to decline MDMA for PTSD treatment is part of a broader, ongoing challenge in integrating psychedelics and empathogens such as MDMA into mainstream medicine, a challenge that is further complicated by the retraction of several key papers in August 2024. These retractions, though necessary to address the issues and shortcomings raised by the FDA’s decision, have led to some disagreement between those conducting the studies, the sponsors, and the researchers who worked on the final papers. One example is the retraction of a 2020 paper in *Psychopharmacology* by Jerome et al.,^71^ which compared a longitudinal pooling of long-term outcomes of six phase-2 trials. The paper was withdrawn by the editors due to “protocol violations amounting to unethical conduct at the MP4 study site by researchers associated with this project”; a decision that was contested by some of the study team, including Dr. Doblin.^72^

Additional papers have been retracted due to concerns about data integrity and methodology—issues that were not flagged during earlier discussions between MAPS and the FDA. These concerns only emerged after the 2024 decision, when the review panel, traditionally focused on standalone pharmaceuticals, grappled with the complexities of assessing substances like MDMA, which are used in conjunction with psychotherapy rather than as isolated treatments.

A 2019 paper by Mithoefer et al., again focusing on study design for the MAPS phase-3 trials, and again retracted by the editors of *Psychoparmacology* for the same reasons as above, demonstrates the seriousness of academic integrity when submitting protocol-oriented papers; the authors, according to the editors, should have removed data from the MP4 site where the misconduct issues were identified. A key point that is raised in the retraction notice, is that the authors were aware of the issues but did not disclose them at the point of submission… wording which the study leads disagree with.^70^

These retractions represent more than isolated instances of flawed research; they underline a deeper problem in the psychedelic research landscape, where the rush to validate MDMA and other substances as therapeutic tools can sometimes outpace the rigorous standards of scientific inquiry. As the FDA’s recent decision shows, the pathway to acceptance for MDMA and other psychedelics in medicine is fraught with challenges, not least of which is ensuring that all data supporting their use is beyond reproach. The credibility of MDMA research is essential not just for regulatory approval but for public trust. The retraction of these studies fuels’ scepticism, both among policymakers and the public, about whether the benefits of MDMA-AT outweigh its risks. Given the decades of research, the successful outcomes, and the profound impact on countless patients and their families, the dedication of committed researchers to bring MDMA into mainstream medicine remains a critical challenge that must be met.

The implications for the future of MDMA as a medicine are significant. Following the FDA’s decision and the retraction of influential studies, the scientific community is faced with the dual burden of rebuilding trust and delivering research that meets the highest ethical and methodological standards. This has broader implications for shifting the paradigm of medicine-focused healthcare, where innovation in treatments like MDMA-AT is often met with resistance. If psychedelic therapies are to gain mainstream acceptance, researchers will need to ensure that their work is bulletproof—free from ethical concerns, bias, or methodological flaws. As Frederick Barrett noted, the field must “double down on the most rigorous methods,” and these retractions only reinforce the need for this recalibration.^68^

Moving forward, researchers like Matthew Johnson, senior researcher at the Center of Excellence for Psilocybin Research and Treatment at Sheppard Pratt, remain hopeful. He acknowledged that while the challenges are significant, the therapeutic value of MDMA will eventually be recognized, provided that future research adheres to the highest ethical and methodological standards (NPR, 2024). Johnson, and many others in the field, believe that MDMA’s unique properties in addressing treatment-resistant PTSD make it an invaluable tool in psychotherapy, but they caution that the path forward will require both patience and precision.

In conclusion, while the FDA’s decision represents a considerable roadblock in the journey toward integrating MDMA into clinical practice, it has also prompted a necessary recalibration for the psychedelic research community. The lessons learned from this setback will hopefully lead to more robust clinical trials and regulatory processes, ensuring that MDMA, and potentially other psychedelics, can be safely and effectively used in therapeutic settings. Despite decades of research, compelling evidence of its therapeutic value, and the urgent need for a paradigm shift in the treatment of behavioral disorders, the future of MDMA-AT remains uncertain. However, with continued research, advocacy, and collaboration among scientists, clinicians, and policymakers, there remains hope that MDMA will eventually find its rightful place in modern medicine.

## Data Availability

No new data were generated or analyzed in support of this research.

## References

[1] Kalant H. The pharmacology and toxicology of “ecstasy” (MDMA) and related drugs. CMAJ. 2001;165(7):917–928. 11599334 PMC81503

[2] Benzenhöfer U, Passie T. Rediscovering MDMA (ecstasy): the role of the American chemist Alexander T Shulgin. Addiction. 2010;105(8):1355–1361. 20653618 10.1111/j.1360-0443.2010.02948.x

[3] Freudenmann RW, Öxler F, Bernschneider-Reif S. The origin of MDMA (ecstasy) revisited: the true story reconstructed from the original documents. Addiction. 2006;101(9):1241–1245. 16911722 10.1111/j.1360-0443.2006.01511.x

[4] Sessa B, Higbed L, Nutt D. A review of 3,4-methylenedioxymethamphetamine (MDMA)-assisted psychotherapy. Front Psychiatry. 2019;10:138. 10.3389/fpsyt.2019.00138 PMC6435835 30949077

[5] Riederer R. The Rebranding of MDMA; 2023.

[6] Mullen F. Schedules of controlled substances. Proposed placement of 3, 4-methylenedioxymethamphetamine into Schedule I. Fed Regist. 1984;49(146):30210–30211.

[7] MDA. Misuse of Drugs Act 1971. London; 1971.

[8] MDA. The Misuse of Drugs Act 1971 (Modification) Order 1977; 1977.

[9] Fisher M. Night lives: Reducing drug-related harm in the night time economy; 2018.

[10] Measham, A., Parker (2001). Dancing on drugs: risk, health and hedonism in the British Club Scene, free association books: 108.

[11] Measham F. ‘3’, in Drug Science and British Drug Policy: Critical Analysis of the Misuse of Drugs Act 1971. Waterside Press; 2022.

[12] Nutt D, King L, Nichols D. New victims of current drug laws. Nat Rev Neurosci. 2013;14:877. doi:10.1038/nrn3530-c2. 24149187

[13] Ricaurte GA, Yuan J, Hatzidimitriou G, Cord BJ, McCann UD. RETRACTED: severe dopaminergic neurotoxicity in primates after a common recreational dose regimen of MDMA ("Ecstasy"). Science. 2002;297(5590):2260–2263. 12351788 10.1126/science.1074501

[14] Passie. The history of MDMA. Oxford University Press; 2023

[15] Parrott AC, Young L. Saturday night fever in ecstasy/MDMA dance clubbers: heightened body temperature and associated psychobiological changes. Temperature. 2014;1(3):214–219. 10.4161/23328940.2014.977182 PMC5008707 27626048

[16] Buchanan D. Leah Betts died 20 years ago and we still can’t be honest about drugs; 2015

[17] UN. Annual prevalence of the use of drugs by region and globally; 2018.

[18] UN. Statistical Annex. P. o. d. u. i. t. g. p. r. a. g. estimates, UNODC; 2024.

[19] Feng LY, Yu WJ, Chang WT, Han E, Chung H, Li JH. Comparison of illegal drug use pattern in Taiwan and Korea from 2006 to 2014. Subst Abuse Treat Prev Policy. 2016;11(1):34 27663984 10.1186/s13011-016-0078-x PMC5034652

[20] Nutt D. New psychoactive substances: Pharmacology influencing UK practice, policy and the law. Br J Clin Pharmacol. 2020;86(3):445–451. doi:10.1111/bcp.14209. 31917863 PMC7080626

[21] Berry J. Remembering the Great British Mephedrone Craze. Vice; 2019.

[22] Nutt DJ. Groundhog decade not brave new world. Drug Sci, Policy Law. 2020;6:205032451989896 doi:10.1177/2050324519898963.

[23] de la Torre R, Farré M, Roset PN, et al. Human Pharmacology of MDMA: Pharmacokinetics, Metabolism, and Disposition. Ther Drug Monit. 2004;26(2):137–144. 15228154 10.1097/00007691-200404000-00009

[24] Holze F, Vizeli P, Müller F, et al. Distinct acute effects of LSD, MDMA, and d-amphetamine in healthy subjects. Neuropsychopharmacology. 2020;45(3):462–471. 31733631 10.1038/s41386-019-0569-3 PMC6969135

[25] Verrico CD, Miller GM, Madras BK. MDMA (Ecstasy) and human dopamine, norepinephrine, and serotonin transporters: implications for MDMA-induced neurotoxicity and treatment. Psychopharmacology. 2007;189(4):489–503. 16220332 10.1007/s00213-005-0174-5

[26] Dunlap LE, Andrews AM, Olson DE. Dark classics in chemical neuroscience: 3,4-methylenedioxymethamphetamine. ACS Chem Neurosci. 2018;9(10):2408–2427. 30001118 10.1021/acschemneuro.8b00155 PMC6197894

[27] Bedi G, Hyman D, de Wit H. Is ecstasy an “empathogen”? Effects of ±3,4-methylenedioxymethamphetamine on prosocial feelings and identification of emotional states in others. Biol Psychiatry. 2010;68(12):1134–1140. 20947066 10.1016/j.biopsych.2010.08.003 PMC2997873

[28] Ball KT, Rebec GV. Role of 5-HT2A and 5-HT2C/B receptors in the acute effects of 3,4-methylenedioxymethamphetamine (MDMA) on striatal single-unit activity and locomotion in freely moving rats. Psychopharmacology. 2005;181(4):676–687. 16001122 10.1007/s00213-005-0038-z

[29] Schenk S, Highgate Q. Methylenedioxymethamphetamine (MDMA): Serotonergic and dopaminergic mechanisms related to its use and misuse. J Neurochem. 2021;157(5):1714–1724. 33711169 10.1111/jnc.15348

[30] Bexis S, Docherty JR. Effects of MDMA, MDA and MDEA on blood pressure, heart rate, locomotor activity and body temperature in the rat involve alpha-adrenoceptors. Br J Pharmacol. 2006;147(8):926–934. 16491100 10.1038/sj.bjp.0706688 PMC2189797

[31] Abraham TT, Barnes AJ, Lowe RH, et al. Urinary MDMA, MDA, HMMA, and HMA excretion following controlled MDMA administration to humans. J Anal Toxicol. 2009;33(8):439–446. 19874650 10.1093/jat/33.8.439 PMC3159864

[32] Best J, Nijhout HF, Reed M. Serotonin synthesis, release and reuptake in terminals: a mathematical model. Theor Biol Med Modell. 2010;7(1):34 10.1186/1742-4682-7-34 PMC2942809 20723248

[33] Mustafa NS, Bakar NHA, Mohamad N, et al. MDMA and the brain: a short review on the role of neurotransmitters in neurotoxicity. Basic Clin Neurosci. 2020;11(4):381–388. 33613876 10.32598/bcn.9.10.485 PMC7878040

[34] Carhart-Harris RL, Wall MB, Erritzoe D, et al. The effect of acutely administered MDMA on subjective and BOLD-fMRI responses to favourite and worst autobiographical memories. Int J Neuropsychopharmacol. 2014;17(4):527–540. 24345398 10.1017/S1461145713001405

[35] Nutt DJ, de Wit H. Putting the MD back into MDMA. Nat. Med.. 2021;27(6):950–951. doi:10.1038/s41591-021-01385-8. 34031606

[36] Ot’alora GM, Grigsby J, Poulter B, et al. 3,4-Methylenedioxymethamphetamine-assisted psychotherapy for treatment of chronic posttraumatic stress disorder: A randomized phase 2 controlled trial. J Psychopharmacol. 2018;32(12):1295–1307. 30371148 10.1177/0269881118806297 PMC6247454

[37] Mithoefer MC, Mithoefer AT, Feduccia AA, et al. 3,4-methylenedioxymethamphetamine (MDMA)-assisted psychotherapy for post-traumatic stress disorder in military veterans, firefighters, and police officers: a randomised, double-blind, doseresponse, phase 2 clinical trial. Lancet Psychiatr. 2018;5(6):486–497. doi:10.1016/s2215-0366(18)30135-4. 29728331

[38] Sessa B, Higbed L, O’Brien S, et al. First study of safety and tolerability of 3,4-methylenedioxymethamphetamine-assisted psychotherapy in patients with alcohol use disorder. J Psychopharmacol. 2021;35(4):375–383. 33601929 10.1177/0269881121991792

[39] Mitchell JM, Bogenschutz M, Lilienstein A, et al. MDMA-assisted therapy for Severe PTSD: a randomized, double-blind, placebo-controlled Phase 3 Study. Nat Med. 2021;27(6):1025–1033. doi:10.1038/s41591-021-01336-3. 33972795 PMC8205851

[40] Mitchell JM, Ot’alora GM, van der Kolk B, et al. MDMA-assisted therapy for moderate to severe PTSD: a randomized, placebo-controlled phase 3 trial. Nat Med. 2023;29(10):2473–2480. 37709999 10.1038/s41591-023-02565-4 PMC10579091

[41] Nardou R, Lewis EM, Rothhaas R, et al. Oxytocin-dependent reopening of a social reward learning critical period with MDMA. Nature. 2019;569(7754):116–120. 30944474 10.1038/s41586-019-1075-9

[42] Sareen J. Posttraumatic stress disorder in adults: impact, comorbidity, risk factors, and treatment. Can J Psychiatry. 2014;59(9):460–467. 25565692 10.1177/070674371405900902 PMC4168808

[43] Gamma A, Buck A, Berthold T, Liechti ME, Vollenweider FX. 3,4-Methylenedioxymethamphetamine (MDMA) modulates cortical and limbic brain activity as measured by [H(2)(15)O]-PET in healthy humans. Neuropsychopharmacology. 2000;23(4):388–395. 10989265 10.1016/S0893-133X(00)00130-5

[44] Weber GF, Johnson BN, Yamamoto BK, Gudelsky GA. Effects of stress and MDMA on Hippocampal gene expression. BioMed Res Int. 2014;2014(1):141396. 24511526 10.1155/2014/141396 PMC3910535

[45] Sapolsky RM, Romero LM, Munck AU. How do glucocorticoids influence stress responses? Integrating permissive, suppressive, stimulatory, and preparative actions. Endocr Rev. 2000;21(1):55–89. 10696570 10.1210/edrv.21.1.0389

[46] Ross JA, Van Bockstaele EJ. The locus coeruleus- norepinephrine system in stress and arousal: unraveling historical, current, and future perspectives. Front Psychiatry. 2021;11:p14 doi:10.3389/fpsyt.2020.601519. PMC7873441 33584368

[47] Parrott AC. Cortisol and 3,4-methylenedioxymethamphetamine: neurohormonal aspects of bioenergetic stress in ecstasy users. Neuropsychobiology. 2009;60(3–4):148–158. doi:10.1159/000253551. 19893332 PMC2826870

[48] Parrott AC, Montgomery C, Wetherell MA, Downey LA, Stough C, Scholey AB. MDMA, cortisol, and heightened stress in recreational ecstasy users. Behav Pharmacol. 2014;25(5–6):458–472. doi:10.1097/fbp.0000000000000060. 25014666

[49] Dumont GJH, Sweep FCGJ, van der Steen R, et al. Increased oxytocin concentrations and prosocial feelings in humans after ecstasy (3,4-methylenedioxymethamphetamine) administration. Soc Neurosci. 2009;4(4):359–366. doi:10.1080/17470910802649470. 19562632

[50] Mithoefer MC, Feduccia AA, Jerome L, et al. RETRACTED “MDMA-assisted psychotherapy for treatment of PTSD: study design and rationale for phase 3 trials based on pooled analysis of six phase 2 randomized controlled trials”. Psychopharmacology. 2019;236(9):2735–2745. 31065731 10.1007/s00213-019-05249-5 PMC6695343

[51] Vizeli P, Liechti ME. Safety pharmacology of acute MDMA administration in healthy subjects. J Psychopharmacol. 2017;31(5):576–588. 28443695 10.1177/0269881117691569

[52] Kamilar-Britt P, Bedi G. The prosocial effects of 3,4-methylenedioxymethamphetamine (MDMA): Controlled studies in humans and laboratory animals. Neurosci Biobehav Rev. 2015;57:433–446. 26408071 10.1016/j.neubiorev.2015.08.016 PMC4678620

[53] Jager G, de Win MML, van der Tweel I, et al. Assessment of cognitive brain function in ecstasy users and contributions of other drugs of abuse: results from an fMRI study. Neuropsychopharmacology. 2008;33(2):247–258. 17460617 10.1038/sj.npp.1301415

[54] Turner JJ, Parrott AC. Is MDMA a human neurotoxin?: diverse views from the discussants. Neuropsychobiology. 2000;42(1):42–48. 10867555 10.1159/000026669

[55] Oehen P, Traber R, Widmer V, Schnyder U. A randomized, controlled pilot study of MDMA (± 3,4-methylenedioxymethamphetamine)-assisted psychotherapy for treatment of resistant, chronic Post-Traumatic Stress Disorder (PTSD). J Psychopharmacol. 2013;27(1):40–52. 23118021 10.1177/0269881112464827

[56] Nutt D, King LA, Saulsbury W, Blakemore C. Development of a rational scale to assess the harm of drugs of potential misuse. Lancet. 2007;369(9566):1047–1053. 17382831 10.1016/S0140-6736(07)60464-4

[57] Nutt DJ, King LA, Phillips LD. Drug harms in the UK: a multicriteria decision analysis. Lancet. 2010;376(9752):1558–1565. doi:10.1016/s0140-6736(10)61462-6. 21036393

[58] Bonomo Y, Norman A, Biondo S, et al. The Australian drug harms ranking study. J Psychopharmacol. 2019;33(7):759–768. 31081439 10.1177/0269881119841569

[59] Crossin R, Cairns R, et al. Australian and New Zealand drug harm rankings: a cross-national comparison. Drug Alcohol Depend. 2020;208:10786831981994

[60] Sessa B, Nutt D. Making a medicine out of MDMA. Br J Psychiatry. 2015;206(1):4–6. 25561485 10.1192/bjp.bp.114.152751

[61] Netzer O, Magal N, Stern Y, et al. (2024). Trauma under psychedelics: MDMA shows protective effects during the peritraumatic period. bioRxiv: 2024.2003.2028.587237.

[62] Gorlin EI, Békés V. Agency via awareness: a unifying meta-process in psychotherapy. Front Psychol. 2021;12:698655. 34335416 10.3389/fpsyg.2021.698655 PMC8316855

[63] Bershad AK, Miller MA, Baggott MJ, de Wit H. The effects of MDMA on socio-emotional processing: Does MDMA differ from other stimulants? J Psychopharmacol. 2016;30(12):1248–1258. 27562198 10.1177/0269881116663120 PMC8753974

[64] Collis H. Ukrainian MP tells MEPs country should be test bed for psychedelics; 2023.

[65] UPRA. Ukrainian Psychedelic Research Association; 2024. https://www.upra.org.ua/en.

[66] Doblin RE, Christiansen M, Jerome L, Burge B. The past and future of psychedelic science: an introduction to this issue. J Psychoact Drugs. 2019;51(2):93–97. 10.1080/02791072.2019.1606472 31132970

[67] Reardon S. MDMA therapy for PTSD rejected by FDA panel; 2024. 10.1038/d41586-024-01622-3 38844808

[68] Stone W. Trouble for ecstasy? What MDMA’s FDA setback could mean for psychedelics; 2024.

[69] Aday JS, Heifets BD, Pratscher SD, Bradley E, Rosen R, Woolley JD. Great Expectations: recommendations for improving the methodological rigor of psychedelic clinical trials. Psychopharmacology. 2022;239(6):1989–2010. doi:10.1007/s00213-022-06123-7. 35359159 PMC10184717

[70] Mithoefer MC, Feduccia AA, Jerome L, et al. Retraction Note: MDMA-assisted psychotherapy for treatment of PTSD: study design and rationale for phase 3 trials based on pooled analysis of six phase 2 randomized controlled trials. Psychopharmacology. 2024; p1. doi:10.1007/s00213-024-06666-x. PMC11513733 39126501

[71] Jerome L, Feduccia AA, Wang JB, Hamilton S, Yazar-Klosinski B, Emerson A, Mithoefer MC, Doblin R. RETRACTED “Long-term follow-up outcomes of MDMA-assisted psychotherapy for treatment of PTSD: a longitudinal pooled analysis of six phase 2 trials”. Psychopharmacology. 2020 237(8):2485–2497. doi:10.1007/s00213-020-05548-2. Retraction in: Psychopharmacology. 2024 Aug 10. doi: 10.1007/s00213-024-06665-y. 32500209 PMC7351848

[72] Jerome L, Feduccia AA, Wang JB, et al. Retraction note: long-term follow-up outcomes of MDMA-assisted psychotherapy for treatment of PTSD: a longitudinal pooled analysis of six phase 2 trials. Psychopharmacology. 2024; p1. doi:10.1007/s00213-024-06665-y. PMC11513715 39126500

